# Argininosuccinate Synthase 1-Deficiency Enhances the Cell Sensitivity to Arginine through Decreased DEPTOR Expression in Endometrial Cancer

**DOI:** 10.1038/srep45504

**Published:** 2017-03-30

**Authors:** Kenji Ohshima, Satoshi Nojima, Shinichiro Tahara, Masako Kurashige, Yumiko Hori, Kohei Hagiwara, Daisuke Okuzaki, Shinya Oki, Naoki Wada, Jun-ichiro Ikeda, Yoshikatsu Kanai, Eiichi Morii

**Affiliations:** 1 Department of Pathology, Osaka University Graduate School of Medicine, Osaka, Japan; 2 Department of Bio-system Pharmacology, Osaka University Graduate School of Medicine, Osaka, Japan; 3 Department of Molecular Genetics, Research Institute for Microbial Diseases, Osaka University, Osaka, Japan; 4 Department of Developmental Biology, Graduate School of Medical Sciences, Kyushu University, Fukuoka, Japan

## Abstract

Argininosuccinate synthetase 1 (ASS1) is a rate-limiting enzyme in arginine biosynthesis. Although ASS1 expression levels are often reduced in several tumors and low ASS1 expression can be a poor prognostic factor, the underlying mechanism has not been elucidated. In this study, we reveal a novel association between ASS1 and migration/invasion of endometrial tumors via regulation of mechanistic target of rapamycin complex (mTORC) 1 signaling. ASS1-knockout cells showed enhanced migration and invasion in response to arginine following arginine starvation. In ASS1-knockout cells, DEPTOR, an inhibitor of mTORC1 signal, was downregulated and mTORC1 signaling was more activated in response to arginine. ASS1 epigenetically enhanced DEPTOR expression by altering the histone methylation. Consistent with these findings, tumor cells at the invasive front of endometrioid carcinoma cases showed lower ASS1 and DEPTOR expression. Our findings suggest that ASS1 levels in each tumor cell are associated with invasion capability in response to arginine within the tumor microenvironment through mTORC1 signal regulation.

Arginine is a non-essential amino acid in humans that is indispensable for the execution of many physiological processes including wound healing, lipid metabolism, hormonal secretion, and activation of reproductive systems^1^,^2^. Arginine is synthesized from citrulline through two sequential enzymatic reactions catalyzed by argininosuccinate synthase (ASS1) and argininosuccinate lyase, in which ASS1 is the rate-limiting enzyme^3^. In the context of cancer cell metabolism, altered amino acid metabolism is important for tumor cell growth^4^,^5^,^6^. The increased use of arginine to fuel anabolic processes is also recognized among the metabolic adaptations of cancer cells, and the endogenous production of arginine is insufficient to meet the demands of rapidly proliferating tumor cells^7^,^8^. Thus, arginine is considered a semi-essential amino acid in certain circumstances such as tumor growth.

The clinical significance of ASS1 has been studied to some extent in several types of human tumor, including breast cancer^9^, myxofibrosarcoma^10^, bladder cancer^11^, and glioblastoma^12^. In these reports, ASS1 deficiency or low ASS1 expression was described as being associated with a poor prognosis for patients. However, the mechanism underlying these findings is not fully understood.

Endometrial cancer arises from the lining of the uterus. Although most patients present with early-stage disease, there is currently little hope for curing patients with advanced stages of endometrial cancer. Regarding metabolism in endometrial cancer, it has been reported that glucose promotes the proliferation and invasion of endometrial cancer cells^13^. However, there have been no reports that examine arginine metabolism in endometrial cancer.

Mechanistic target of rapamycin (mTOR) is a serine/threonine kinase, which exists in two complexes: mTORC1 and mTORC2, and its signaling pathway plays a central role in physiological cell growth and survival control^14^. Tumor cell adhesion, motility, and invasion capability are also regulated by mTORC1 and mTORC2^15^,^16^. Their kinase activities are negatively regulated by DEPTOR, which is a recently identified mTOR binding protein^17^. DEPTOR has antitumor activity in pancreatic cancer^18^, esophageal cancer^19^, and lung cancer^20^, whereas DEPTOR promotes the survival of myeloma cells^17^,^21^ and cervical squamous cell carcinoma cells^22^. It is known that amino acids, particularly arginine, leucine, and glutamine, activate mTORC1^23^,^24^,^25^. It has recently been reported that arginine regulates mTORC1 activity by inducing its recruitment to lysosomal membranes^26^. In addition, SLC38A9 is a putative lysosomal arginine sensor^26^ and CASTOR1 is a cytosolic arginine sensor^27^,^28^. Although it is well known that arginine stimulates mTORC1 activity, the involvement of ASS1 and arginine that has been endogenously synthesized by ASS1 in the mTORC1 signaling pathway has not been elucidated.

Here, we present a novel pathological role of ASS1 in tumor cells. ASS1-KO endometrial cancer cells generated by the Clustered Regularly Interspaced Short Palindromic Repeats (CRISPR)/CRISPR-associated 9 (CRISPR/Cas9) system showed enhanced cell sensitivity to arginine and resulted in increased cell motility and invasion capability in response to arginine following arginine starvation. Further molecular analysis revealed that ASS1-KO cells showed lower DEPTOR expression, resulting in faster and higher mTORC1 activation when re-supplemented with arginine following arginine starvation. It was also shown that ASS1 positively regulated DEPTOR expression by altering histone methylation. Consistent with these *in vitro* results, immunohistochemistry using human endometrioid carcinoma clinical specimens demonstrated that cancer cells at the tumor invasive front showed lower ASS1 and DEPTOR expression, and higher ribosomal protein S6 phosphorylation (pS6) than those in the center of the tumor. Thus, our findings provide novel evidence for the importance of ASS1 in the migration/invasion capability of tumor cells, which might be helpful for understanding the pathological significance of arginine metabolism in tumor cells.

## Result

### Absence of ASS1 has no influence on growth, motility, and invasion of the human endometrial cancer cell lines cultured in arginine-replete conditions

**Figure 1:**
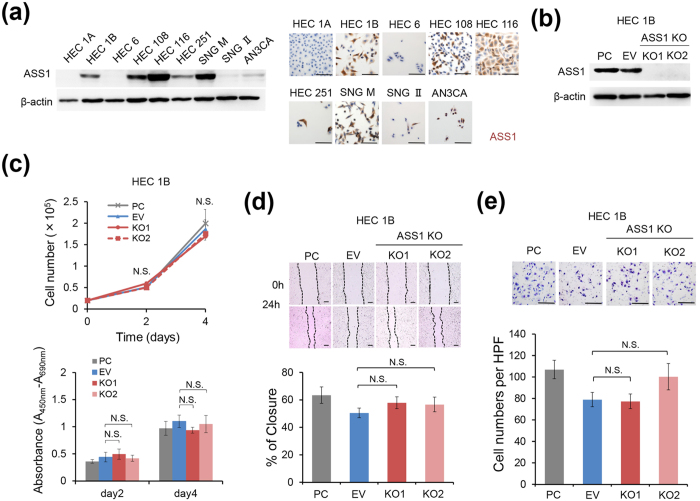
ASS1 deficiency does not affect proliferation, motility, and invasion of HEC1B cells when cultured in DMEM containing arginine. (**a**) Immunoblotting and immunocytochemistry of ASS1 in endometrial cancer cell lines. Scale bars, 100 μm. (**b**) Immunoblotting of ASS1 in PC, EV, and ASS1-KO HEC1B cells generated using the CRISPR/Cas9 system. (**c**–**e**) When PC, EV, and ASS1-KO HEC1B cells were cultured in DMEM prepared for standard culture conditions (complete DMEM), there was no significant difference in (**c**) cell proliferation (n = 3) assessed by cell counting (upper panel) and WST-1 assay (lower panel). A total of 2 × 10^4^ cells were seeded into a 6-well plate for the cell counting assay and 1 × 10^3^ cells were seeded into a 96-well plate for the WST-1 assay. Data are representative of three independent experiments. (**d**) Cell motility (n = 3) assessed using a scratch assay, and (**e**) invasion ability (n = 3) assessed using a Transwell Matrigel invasion assay. Data are representative of three independent experiments. Data are shown as mean ± SD. Scale bars, 400 μm in (**d**), 200 μm in (**e**). N.S., not significant, Student’s t-test. Full-length blots are presented in Supplementary Fig. S12.

First, we examined the expression levels of ASS1 in endometrial cancer cell lines by immunoblotting and immunocytochemistry. As shown in Fig. 1(a), several endometrial cancer cell lines showed sufficient levels of ASS1 expression. To examine the significance of ASS1 in endometrial cancer cells, we disrupted the *ASS1* gene in HEC1B and AN3CA cells, which showed ASS1 expression among the endometrial cancer cell lines, using the CRISPR/Cas9 system and successfully established ASS1-knockout (ASS1-KO) HEC1B and AN3CA cells (Fig. 1(b) and see Supplementary Fig. S1(a), S1(b), S1(c) and S2(a)). There was no significant difference in proliferation between ASS1 wild-type parental cells (PC), empty vector-transfected cells (EV), and independent clones of ASS1-KO cells of HEC1B or AN3CA cells (Fig. 1(c) and see Supplementary Fig. S2(b)). In addition, an *in vitro* scratch assay and an invasion assay using the Transwell filter system with Matrigel demonstrated that depletion of ASS1 in HEC1B and AN3CA cells did not affect their motility and invasion capability (Fig. 1(d) and see Supplementary Fig. S2(c) and S2(d)). Thus, the absence of ASS1 expression had no influence on *in vitro* cell growth, cell motility, and invasion capability, at least when these cells were cultured in Dulbecco’s modified Eagle’s medium (DMEM) conditioned for standard cell culture, which is arginine-replete.

### ASS1 is involved in tumor cell motility and invasion capability when cells are re-supplemented with arginine following arginine starvation

Since cells can utilize extracellular arginine by transporting it through the cationic amino acid transporters^29^, we hypothesized that ASS1 does not influence tumor cell behavior under a sufficient supply of extracellular arginine. To test this hypothesis, we performed *in vitro* experiments under arginine-depleted conditions. Importantly, we demonstrated that the proliferation of HEC1B cells declined when the extracellular arginine concentration was reduced, especially under 80 μM (see Supplementary Fig. S3). This supports the idea that tumor cells are highly dependent on extracellular arginine.

**Figure 2:**
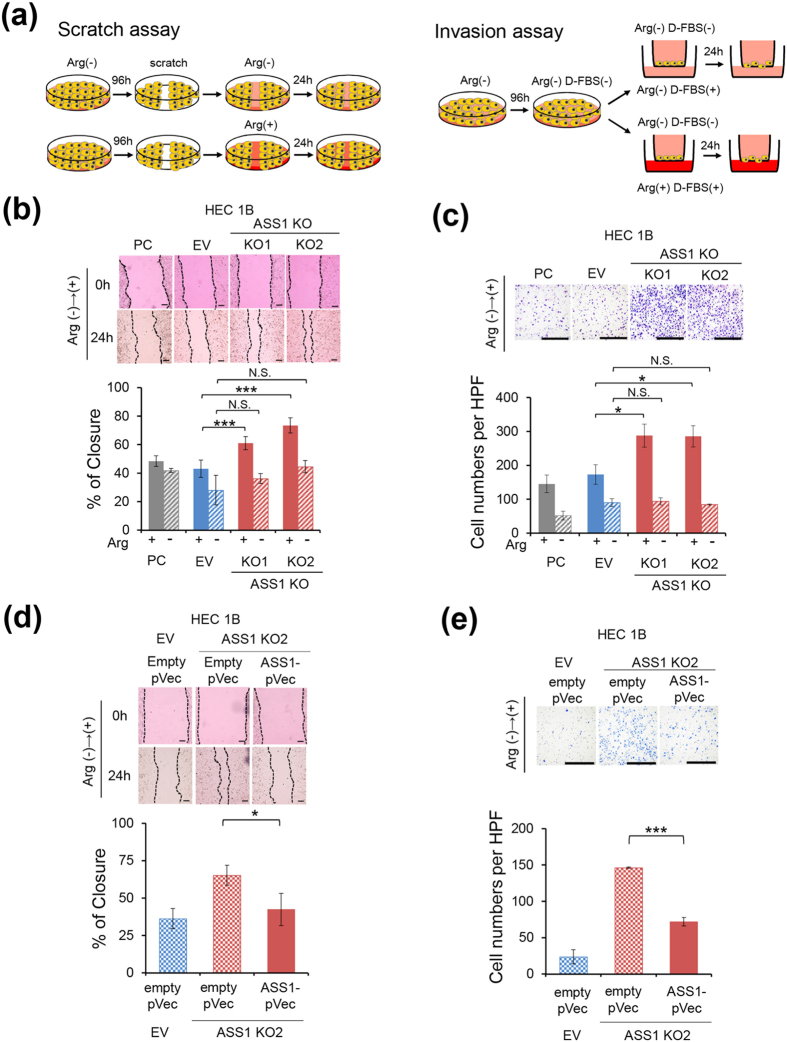
ASS1 deficiency enhances the cell motility and invasion capability of HEC1B cells in response to re-supplementation with arginine following arginine starvation. (**a**) Schematic showing the experimental procedures of the *in vitro* scratch assay and invasion assay following 96-hour arginine starvation. (**b**) and (**c**) Cell motility or invasion capability of PC, EV, or ASS1-KO HEC1B cells was assessed by (**b**) scratch assay (n = 3) or (**c**) Transwell Matrigel invasion assay (n = 3). Data are representative of four independent experiments. (**d** and **e**) ASS1-KO HEC1B cells were transiently transfected with a plasmid construct expressing ASS1 (ASS1-pVec) or an empty control vector (empty pVec). 12 hours after transfection, the medium was replaced by arginine-depleted DMEM with 10% dialyzed FBS. After 96 hours of arginine starvation, these cells were subjected to (**d**) scratch assay (n = 3) or (**e**) Transwell Matrigel invasion assay (n = 3). Data are representative of three independent experiments. Data are shown as the mean ± SD. Scale bars, 400 μm in (**b**) and (**d**), 200 μm in (**c**) and (**e**). *p < 0.05, **p < 0.01, ***p < 0.001, Student’s t-test.

We first compared the proliferation of PC, EV, and ASS1-KO cells of HEC1B or AN3CA cells, but there was no significant difference in arginine-depleted DMEM (see Supplementary Fig. S4). We next evaluated cell motility and invasion capability under arginine-depleted conditions. PC, EV, and ASS1-KO cells of HEC1B or AN3CA cells were cultured for 96 hours in arginine-depleted DMEM, followed by re-supplementation with arginine (final concentration 400 μM), and then subjected to an *in vitro* scratch assay and invasion assay (Fig. 2(a)). Whereas no significant difference was found without arginine re-supplementation, ASS1-KO cells showed higher cell motility than PC and EV cells when re-supplemented with arginine following arginine starvation in HEC1B and AN3CA cells (Fig. 2(b) and see Supplementary Fig. S5(a)). Notably, the increased cell motility of ASS1-KO HEC1B cells was not observed when these cells underwent starvation and then were re-supplemented with lysine, which is a cationic amino acid like arginine and also an essential amino acid (see Supplementary Fig. S6(a) and S6(b)), suggesting that the specific activity of arginine affects tumor cell motility and invasion capability. Similar to the results from the scratch assay, the *in vitro* invasion assay using a Transwell filter system with Matrigel demonstrated that ASS1-KO cells showed higher invasion capability than PC and EV cells when arginine was re-supplemented to the lower chamber of the Transwell (final concentration 400 μM) in HEC1B and AN3CA cells. However, no difference was found when arginine was not re-supplemented (Fig. 2(c) and see Supplementary Fig. S5(b)). In addition, enhanced cell motility and invasion capability of ASS1-KO HEC1B cells in response to arginine re-supplementation following arginine starvation was prevented by exogenous ASS1 expression, excluding off-target effects of genome editing (Fig. 2(d) and (e)). These results indicate that, under arginine-depleted conditions, the absence of ASS1 enhances cell motility and invasion capability in response to re-supplementation with arginine.

### Decreased DEPTOR expression is involved in enhanced cell motility and invasion capability of ASS1-KO cells

**Figure 3:**
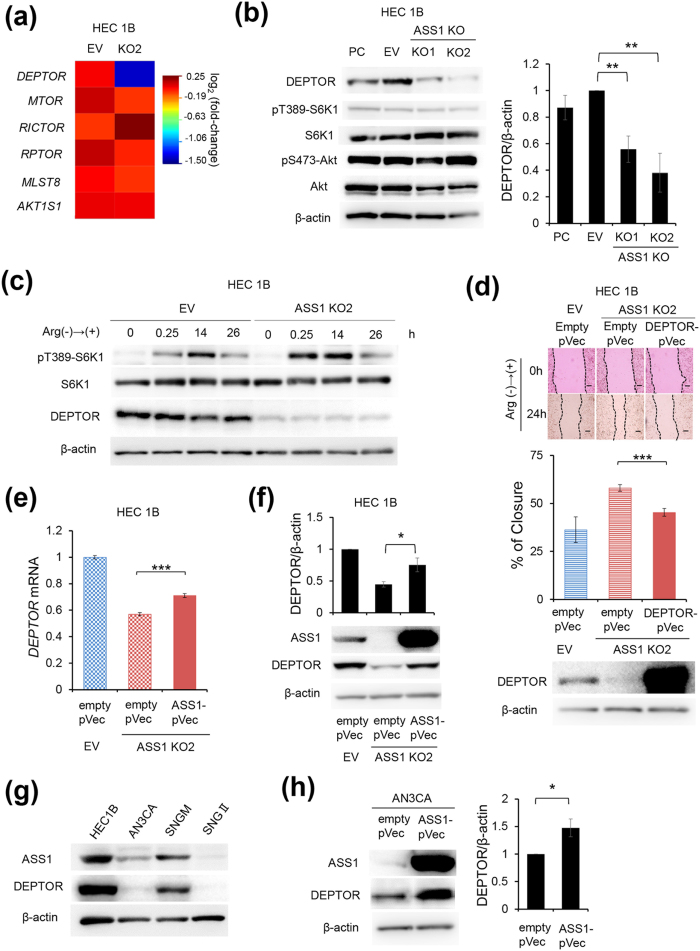
Enhanced sensitivity of ASS1-KO HEC1B cells to arginine is due to down-regulated expression of DEPTOR and resultant enhanced mTORC1 activity. (**a**) The expression profile of genes related to the mTOR complex from the results of the DNA microarray (n = 2). (**b**) Representative immunoblotting of PC, EV, and ASS1-KO HEC1B cells cultured in arginine-replete conditions. DEPTOR: β-actin ratios were calculated based on band intensities measured with densitometry. Data are shown as mean ± SD from four independent experiments, analyzed by one-sample t-tests. **p < 0.01. (**c**) Representative immunoblotting of the mTORC1 signaling pathway and DEPTOR in EV and ASS1-KO HEC1B cells cultured in arginine-depleted conditions for 96 hours, followed by re-supplementation with arginine (n = 3). (**d**) ASS1-KO HEC1B cells were transiently transfected with a plasmid construct expressing DEPTOR (DEPTOR-pVec) or an empty control vector (empty pVec). 12 hours after transfection, the medium was replaced by arginine-depleted DMEM with 10% dialyzed FBS. After 96 hours of arginine starvation, these cells were subjected to a scratch assay (n = 3) followed by re-supplementation with arginine. EV HEC1B cells transfected with an empty were used as a control. Data are shown as mean ± SD. Scale bars, 400 μm. ***p < 0.001, Student’s t-test. Data are representative of three independent experiments. (e and f) ASS1-KO HEC1B cells were transiently transfected with DEPTOR-pVec or empty pVec. 72 hours after transfection, (**e**) *DEPTOR* mRNA and (**f**) DEPTOR protein expression were evaluated by real-time PCR and immunoblotting respectively. Data are representative of three independent experiments. ***p < 0.001, Student’s t-test. In (**f**) immunoblotting, DEPTOR: β-actin ratios were calculated based on band intensities measured with densitometry. Data are shown as mean ± SD from three independent experiments, analyzed by Student’s t-tests. *p < 0.05 (**g**) Representative immunoblotting of ASS1 and DEPTOR in indicated endometrial cancer cell lines (n = 2). (**h**) AN3CA cells were transiently transfected with ASS1-pVec or empty pVec. 72 hours after transfection, these cells were lysed and subjected to immunoblotting of ASS1 and DEPTOR. DEPTOR: β-actin ratios were calculated based on band intensities measured with densitometry. Data are shown as mean ± SD from three independent experiments, analyzed by one-sample t-tests. *p < 0.05. Full-length blots are presented in Supplementary Fig. S12 and S13.

To elucidate the mechanisms underlying the enhanced sensitivity to arginine in the absence of ASS1 expression in endometrial cancer cells, we performed DNA microarray-based gene expression profiling using EV and ASS1-KO HEC1B cells. Since accumulating evidence has shown that arginine regulates mTORC1 activity^23^,^26^, we focused on the expression patterns of genes related to the mTOR complex. As shown in Fig. 3(a), ASS1-KO HEC1B cells had lower *DEPTOR* transcript abundance than EV HEC1B cells, while no significant difference was observed in the expression levels of genes coding for other mTOR complex components such as *mTOR, RAPTOR*, or *RICTOR*. ASS1-KO AN3CA cells had also lower *DEPTOR* transcript abundance than EV AN3CA cells (see Supplementary Fig. S7(a)). Indeed, immunoblot analysis confirmed that the expression level of DEPTOR protein was significantly decreased in ASS1-KO cells compared with that in PC and EV cells, which were cultured in arginine-replete conditions in HEC1B and AN3CA cells (Fig. 3(b) and see Supplementary Fig. S7(b)). However, there were no significant differences between PC, EV, and ASS1-KO HEC1B cells in ribosomal protein S6 kinase 1 (S6K1) and Akt phosphorylation, which represent mTORC1 and mTORC2 activities, respectively (Fig. 3(b)). In addition, when cultured in arginine-depleted conditions, ASS1-KO HEC1B cells still showed decreased DEPTOR expression compared with PC and EV HEC1B cells, but no significant differences were found in mTORC1 and mTORC2 activities (see Supplementary Fig. S8).

Since DEPTOR is a negative regulator of the mTOR complex, it was speculated that decreased DEPTOR expression in ASS1-KO cells could affect cell behavior only when cells are under specific conditions, for example, when mTORC1 signaling is momentarily induced in response to rapidly increased arginine concentration. We thus cultured EV and ASS1-KO HEC1B cells in arginine-depleted DMEM for 96 hours, and subsequently replaced the medium with DMEM containing 400 μM arginine. At 0.25, 14, and 26 hours after medium replacement, cell lysates were prepared from these cells and mTORC1 activation was assessed by immunoblotting against phosphorylated S6K1. While S6K1 phosphorylation in EV HEC1B cells reached a peak 14 hours after arginine re-supplementation, ASS1-KO HEC1B cells showed higher S6K1 phosphorylation 0.25 hours after re-supplementation (Fig. 3(c)). These findings indicated that ASS1-KO HEC1B cells showed faster and higher mTORC1 activation than EV HEC1B cells in response to the re-supplementation with arginine following arginine starvation. The addition of the mTOR inhibitor Torin1 prevented the higher cell motility of ASS1-KO HEC1B cells (see Supplementary Fig. S9). Furthermore, exogenous DEPTOR expression prevented the enhanced cell motility of ASS1-KO HEC1B cells in response to arginine re-supplementation after arginine starvation, supporting the idea that enhanced cell motility of ASS1-KO cells is due to DEPTOR-dependent mechanisms (Fig. 3(d)).

Based on these findings, it was determined that ASS1 is a positive regulator of DEPTOR expression. Indeed, ASS1-KO HEC1B cells recovered DEPTOR mRNA and protein expression levels when transfected with a plasmid construct expressing ASS1 (Fig. 3(e) and (f)). This result eliminates the possibility that decreased DEPTOR expression was due to off-target effects of genome editing.

Moreover, we examined ASS1 and DEPTOR expression levels and their correlation among several endometrial cancer cell lines by immunoblotting. These cell lines showed a positive correlation between ASS1 and DEPTOR expression levels (Fig. 3(g)). Consistent with this result, the expression level of DEPTOR in AN3CA cells was significantly increased in proportion to the increased ASS1 expression induced by transfection of a plasmid construct expressing ASS1 (Fig. 3(h)). Taken together, these results show that ASS1 is a positive regulator of DEPTOR expression, and that the absence of ASS1 causes faster and higher mTORC1 activation, resulting in enhanced motility and invasion capability in endometrial cancer cells.

### ASS1 positively regulates DEPTOR expression by altering histone methylation

**Figure 4:**
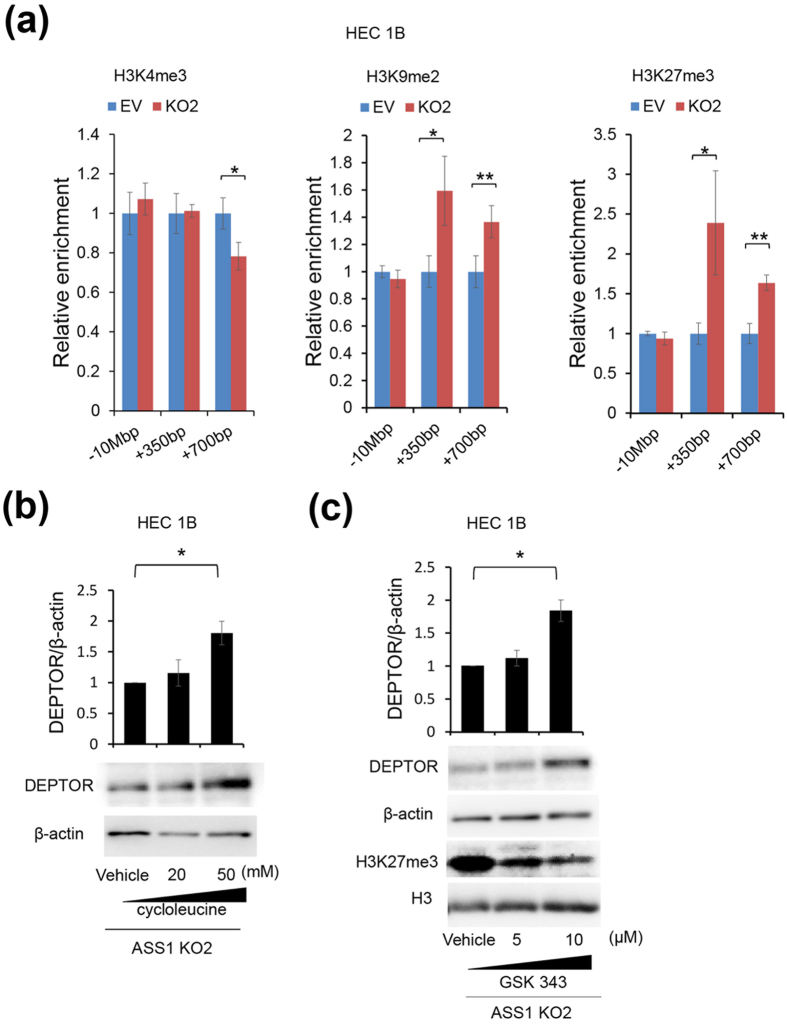
ASS1 positively regulates DEPTOR expression by altering histone methylation in the *DEPTOR* promoter region. (**a**) ChIP-qPCR analysis of the 5′ regulatory region of DEPTOR in EV and ASS1-KO HEC1B cells using antibodies to H3K4me3, H3K9me2, and H3K27me3 (n = 3). The locations of the qPCR primers are relative to the transcriptional start site of DEPTOR. “+” and “−” means downstream and upstream from the transcriptional start site of DEPTOR, respectively. −10 Mbp was set as a negative control. Data are expressed as a ratio relative to the percent of input of EV. Data are representative of three independent experiments. Data are shown as mean ± SD. *p < 0.05, **p < 0.01. Student’s t-test was applied other than H3K27me3 + 350 bp which was analyzed by Welch’s t-test. (**b**) ASS1-KO HEC1B cells (5 × 10^5^ cells) were resuspended in 3 ml of DMEM and seeded into a 6-well plate. Twelve hours after seeding, medium was replaced with DMEM containing the indicated concentration of cycloleucine. Forty-eight hours after medium replacement, cells were lysed and analyzed by immunoblotting for DEPTOR. Data are representative of three independent experiments. DEPTOR: β-actin ratios were calculated based on band intensities measured with densitometry. Data are shown as mean ± SD from three independent experiments, analyzed by one-sample t-tests. *p < 0.05. (**c**) ASS1-KO HEC1B cells (2 × 10^5^ cells) were resuspended in 3 ml of DMEM and seeded into a 6-well plate. Twelve hours after seeding, medium was replaced with DMEM containing the indicated concentration of GSK343. 72 hours after medium replacement, cells were lysed and analyzed by immunoblotting. Data are representative of three independent experiments. DEPTOR: β-actin ratios were calculated based on band intensities measured with densitometry. Data are shown as mean ± SD from three independent experiments, analyzed by one-sample t-tests. *p < 0.05. Full-length blots are presented in Supplementary Fig. S13.

Arginine is a substrate for creatine synthesis. S-adenosylmethionine (SAM), which is a universal donor for the methylation reaction that modifies DNA, histones, and other proteins, is also a substrate for creatine synthesis. The use of SAM for creatine synthesis has been considered a major SAM-consuming reaction^30^. Therefore, we hypothesized that ASS1 might reduce methylation by consuming SAM through arginine and creatine synthesis. To test this hypothesis, we evaluated DNA methylation within CpG islands at the *DEPTOR* promoter region of EV and ASS1-KO HEC1B cells, but no significant difference was found in DNA methylation at the *DEPTOR* promoter region between EV and ASS1-KO HEC1B cells (see Supplementary Fig. S10). We then examined the histone methylation at the *DEPTOR* promoter region of EV and ASS1-KO cells of HEC1B or AN3CA cells by chromatin immunoprecipitation (ChIP) assays. As expected, ChIP assays showed increased dimethylation of H3 lysine 9 (H3K9me2) and trimethylation of H3 lysine 27 (H3K27me3), which are repressive chromatin marks, in the *DEPTOR* promoter region of ASS1-KO HEC1B cells (Fig. 4(a)). H3K27me3 in the *DEPTOR* promoter region of ASS1-KO AN3CA cells was also increased (see Supplementary Fig. S11). Trimethylation of H3 lysine 4 (H3K4me3), an epigenetic mark that is associated with transcriptionally active chromatin, was also reduced in the *DEPTOR* promoter region of ASS1-KO HEC1B cells, but the degree of reduction was relatively low. Furthermore, DEPTOR expression in ASS1-KO HEC1B cells was increased by treatment with cycloleucine, which is an inhibitor of SAM synthesis, or with GSK343, which inhibits histone H3K27 trimethylation, supporting the idea that the ASS1-mediated regulation of DEPTOR expression is due to alteration of histone methylation (Fig. 4(b) and (c)). Thus, it was demonstrated that ASS1 positively regulated DEPTOR expression by altering histone methylation, but not DNA methylation.

### Expression patterns of ASS1 in clinical samples of human endometrial cancers

**Figure 5:**
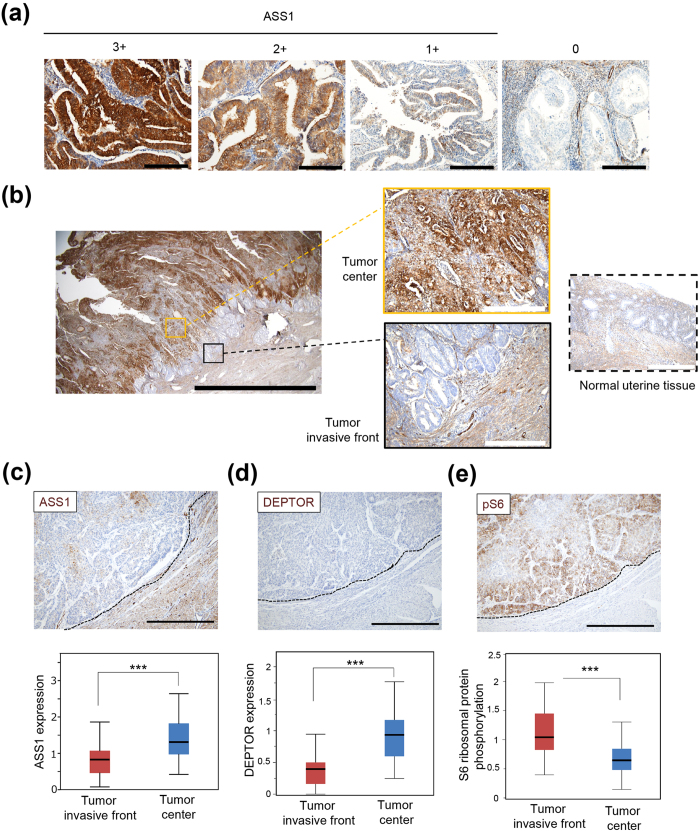
Expression pattern of ASS1 and mTOR-associated molecules in clinical specimens derived from human endometrial cancer tissues. (**a**) Representative images of immunohistochemistry of ASS1 using endometrioid carcinoma tissues with high (score 3), intermediate (score 2), low (score 1), or null (score 0) signal intensity, which were used for calculating H-scores. Scale bars, 200 μm. (**b**) Representative image of immunohistochemistry for ASS1, in which the representative boxed areas of the tumor center, tumor invasive front and normal uterine tissue are enlarged. Black scale bar, 5 mm. White scale bars, 500 μm. (**c**–**e**) ASS1, DEPTOR protein expression levels or ribosomal protein S6 phosphorylation (pS6) were statistically analyzed by comparing H-scores calculated from four fields at 200x magnification in the tumor invasive front or tumor center from 74 (ASS1) or 74 (DEPTOR and pS6) cases, respectively. Scale bars, 500 μm. ***p < 0.001, Welch’s t-test.

Finally, we examined ASS1 expression in 74 cases of endometrioid carcinoma clinical specimens by immunohistochemistry. The clinicopathological characteristics are shown in Supplementary Table S1. As shown in Fig. 5(a), various levels of ASS1 expression were found among these specimens. Interestingly, tumor cells at the tumor invasive front showed lower ASS1 expression than those in the tumor center (Fig. 5(b) and (c)). As described above, *in vitro* experiments demonstrated that ASS1-KO cells showed higher cell motility and invasion capability than PC and EV cells when re-supplemented with arginine following arginine starvation in HEC1B and AN3CA cells. Based on these findings, it was suggested that the characteristic ASS1 expression pattern at the tumor invasive front in endometrioid carcinoma specimens is the result of enhanced invasion capability of tumor cells with decreased ASS1 expression. In addition, DEPTOR expression levels were lower at the tumor invasive front than in the tumor center, which was similar to the expression pattern of ASS1. Ribosomal protein S6 phosphorylation levels showed a complementary pattern to ASS1 (Fig. 5(d) and (e)). These findings were consistent with the results of *in vitro* experiments showing that the enhanced cell sensitivity to arginine under arginine-depleted conditions in ASS1-KO cells was due to decreased DEPTOR expression. Thus, in human endometrial cancer, tumor cells at the tumor invasive front, which had a low abundance of ASS1, showed lower DEPTOR expression and higher mTORC1 activation than those in the tumor center. These results suggested that, even *in vivo*, absence of ASS1 may enhance cell motility and invasion capability through decreased DEPTOR expression.

## Discussion

**Figure 6:**
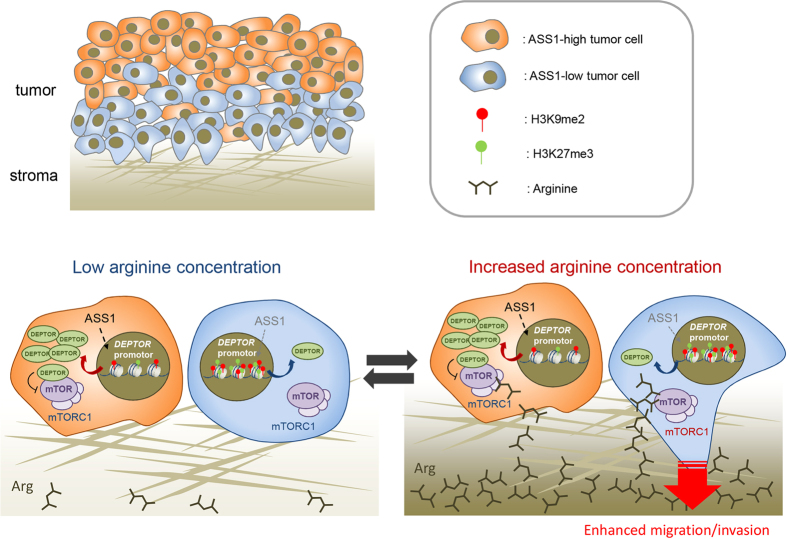
Schematic presentation of the mechanism of increased migration/invasion capability of tumor cells with low ASS1 expression in response to arginine concentration change. When the tumor microenvironment contains a low concentration of arginine, tumor cells do not show remarkable mTORC1 activation regardless of ASS1 expression levels. When arginine concentration rapidly rises in the tumor microenvironment, tumor cells with low ASS1 expression show faster and higher arginine-induced mTORC1 activation and resultant enhanced invasiveness because of their decreased DEPTOR expression via alteration of histone methylation. Tumor cells with high ASS1 expression, however, are unaffected. The arginine concentration in the tumor microenvironment is expected to fluctuate according to the nutritional status. In response to these sequential fluctuations of arginine concentration in the tumor microenvironment, especially in the stroma, tumor cells with low ASS1 expression might show a tendency to invade ahead of ASS1-expressing tumor cells and accumulate along the invasive frontline of the tumor.

In this study, we found a novel association between ASS1 and tumor cell properties of endometrial cancer. Notably, it was shown that ASS1-deficient endometrial cancer cells showed enhanced migration and invasion capability in response to increased arginine concentration due to suppressed DEPTOR expression. DEPTOR expression is epigenetically regulated by changes in histone methylation (Fig. 6).

First, we generated ASS1-KO cells using the CRISPR/CAS9 system and conducted *in vitro* functional assays. Interestingly, ASS1-KO cells showed a higher invasion capability when arginine was re-supplemented in the lower chamber of a Transwell filter system with Matrigel following arginine starvation. PC, EV, and ASS1-KO cells, however, showed no significant differences in proliferation, cell motility, and invasion capability when cultured in arginine-replete conditions. These results suggest that ASS1 might influence tumor migration and invasion when the concentration of extracellular arginine is drastically increased from very low levels to sufficient levels. From these results, we had speculated that arginine could act like a chemoattractant especially for ASS1-KO cells. In the context of the *in vivo* tumor microenvironment, the ASS1 expression levels of each tumor cell may correlate with its invasion capability in response to changes in arginine concentration within the tumor microenvironment. In particular, it is known that blood arginine levels change according to food intake^31^, suggesting that arginine concentration may constantly fluctuate in and around vessels of the tumor microenvironment. In such conditions, tumor cells with decreased ASS1 expression near the stroma may be attracted to arginine and invade ahead of other ASS1-expressing tumor cells. The results of immunohistochemistry using endometrioid carcinoma clinical specimens, in which tumor cells with decreased ASS1 expression lined up along the stroma, are consistent with this hypothesis.

Next, we revealed that ASS1 influenced the mTORC1 signaling pathway. While there was no significant difference between PC, EV, and ASS1-KO HEC1B cells in mTORC1 and mTORC2 activities when cultured in arginine-replete or arginine-depleted conditions, ASS1-KO HEC1B cells showed faster and higher arginine-induced mTORC1 activation when re-supplemented with arginine following arginine starvation (Fig. 3(b,c), and see Supplementary Fig. S8). Further studies revealed that ASS1 positively regulated DEPTOR expression by altering the histone methylation. The amounts of H3K9me2 and H3K27me3, which are repressive chromatin marks, at the DEPTOR promoter region were augmented in ASS1-KO cells compared with those in EV cells (Fig. 4(a) and see Supplementary Fig. S11). These results suggest that, through decreased expression of DEPTOR, due to changes in histone methylation, ASS1-KO cells showed faster and higher arginine-induced short-term mTORC1 activation and showed increased cell motility and invasion capability when re-supplemented with arginine following arginine starvation. It is also possible that an inhibitory role of DEPTOR on mTORC1 activation might not be apparent when the arginine concentration is unchanged, but might become apparent only when the extracellular arginine concentration is rapidly elevated.

Recently, there have been several reports suggesting an association between arginine and the mTORC1 signaling pathway. In particular, arginine is sensed by CASTOR in the cytosol and SLC38A9 at the lysosome membrane, inducing mTORC1 recruitment to the lysosomal membrane and activating its signaling pathway^26^,^27^,^28^. Thus, it is well known that arginine, which is transported into cells from the extracellular environment, stimulates mTORC1 activity. However, our findings indicate that ASS1, which contributes to *de novo* arginine synthesis, is also involved in regulating mTORC1 activity.

So far, proteins such as che-1^21^, cMAF^17^, and Baf60c-Six4^32^ have been reported as factors involved in DEPTOR transcription. DEPTOR is degraded via the ubiquitin-proteasome pathway by mTOR-dependent phosphorylation^33^. Thus, DEPTOR expression is strictly regulated. Our study demonstrated that ASS1 regulated DEPTOR expression by altering histone methylation at the DEPTOR promoter region. It has been recently reported that SAM, a universal donor for the methylation reaction, is largely consumed through creatine synthesis in which arginine is also a substrate^30^ and that SAM consumption during creatine synthesis reduces the histone methylation^34^. Our study showed that the amounts of H3K9me2 and H3K27me3 at the DEPTOR promoter region were reduced in EV cells compared with those in ASS1-KO cells (Fig. 4(a) and see Supplementary Fig. S11). This result suggests the possibility that arginine, which was synthesized *de novo* by ASS1, consumed SAM through creatine synthesis and reduced repressive histone methylation at the DEPTOR promoter region, resulting in positively regulated DEPTOR expression. However, in our study, we did not elucidate why ASS1 regulated epigenetic states specifically at the DEPTOR promoter region.

It has been reported that ASS1 knockdown in bladder cancer^11^ and myxofibrosarcoma^10^ cell lines and DEPTOR knockout in esophageal squamous cell carcinoma^19^ and lung adenocarcinoma^20^ cell lines showed increased cell motility and invasion ability, respectively. These findings suggest that the association between ASS1 and DEPTOR that we found in this study might be applicable to other types of tumor. Furthermore, several studies using clinical samples of breast cancer^9^, myxofibrosarcoma^10^, bladder cancer^11^, and glioblastoma^12^ showed that ASS1 deficiency or low ASS1 expression is a poor prognostic factor. The association between ASS1, DEPTOR, and mTORC1 activity could help us to obtain a better understanding of these results.

In conclusion, we demonstrated that endometrial cancer cells with decreased ASS1 expression show increased cell motility and invasion capability in response to changes in arginine concentration and tend to invade toward stroma, ahead of other ASS1-expressing cells. This is due to suppressed DEPTOR expression as a result of altered histone methylation. These findings provide novel evidence for the pathological significance of arginine metabolism within tumor cell heterogeneity and for the involvement of ASS1 in tumor cell properties.

## Methods

### Antibodies

Antibodies were obtained from the following sources: rabbit polyclonal antibody to ASS1 (HPA020896) was from Sigma-Aldrich (Milwaukee, WI); mouse monoclonal antibodies to ASS1 (ab124465), H3K9me2 (ab1220), and H3K27me3 (ab6002) were from Abcam (Cambridge, UK); rabbit monoclonal antibodies to p70 S6 Kinase (#2708), Phospho-p70 S6 Kinase (Thr389) (#9234), Phospho-S6 Ribosomal Protein (Ser240/244) (#5364), Akt (#4691), Phospho-Akt (Ser473) (#4060), DEPTOR/DEPDC6 (#11816), H3K4me3 (#9751), Histone H3 (#4620 and #4499) and β-Actin (HRP-conjugated) (#5125) were from Cell Signaling Technology (Danvers, MA); and anti-mouse (#330) and anti-rabbit (#458) secondary antibodies were from Medical & Biological Laboratories (Nagoya, Japan).

### Plasmids

Human ASS1 and DEPTOR cDNA constructs were obtained from OriGene (Rockville, MD). Plasmid SC110830 was used as a vector for ASS1 (pCMV6-XL4-ASS1) and plasmid SC112427 was used as a vector for DEPTOR (pCMV6-XL4-DEPTOR).

### Reagents

Reagents were obtained from the following sources: Puromycin was from Merck Millipore (Bedford, MA); Torin1 was from Cell Signaling Technology; Cycloleucine and GSK343 were from Sigma-Aldrich; L-arginine hydrochloride, L-lysine hydrochloride, and L-glutamine were from Wako (Osaka, Japan); Dialyzed Fetal Bovine Serum (FBS) and glutamine-, lysine-, and arginine-free DMEM were from Thermo Fisher Scientific (Waltham, MA). Arginine-depleted DMEM was prepared by adding L-glutamine (4 mM) and L-lysine hydrochloride (800 μM) to glutamine-, lysine-, and arginine-free DMEM. Lysine-depleted DMEM was prepared by adding L-glutamine (4 mM) and L-arginine hydrochloride (400 μM) to glutamine-, lysine-, and arginine-free DMEM.

### Cell lines and cell culture

For culture in standard conditions (arginine-replete), cell lines were cultured in DMEM supplemented with 10% FBS, penicillin (100 IU/mL), and streptomycin (100 μg/mL) and maintained at 37 °C in 5% CO_2_. In arginine or lysine depletion experiments, cells were cultured in arginine- or lysine-depleted DMEM supplemented with 10% dialyzed FBS, penicillin (100 IU/mL), and streptomycin (100 μg/mL) and maintained at 37 °C in 5% CO_2_.

### Patients

All experimental protocols were approved by the Ethical Review Board of the Graduate School of Medicine, Osaka University (No. 15234) and were performed in accordance with the Committee guidelines and regulations. The informed consent was obtained from all patients. We examined 74 patients undergoing surgery for endometrioid cancer of the uterine corpus at Osaka University Hospital from 2010 to 2015. Resected specimens were fixed in 10% formalin and processed for paraffin embedding. Specimens were stored at room temperature in a dark room. Specimens to be used for evaluation were sectioned at 4-μm thickness and stained with HE.

### Immunohistochemistry

Paraffin-embedded tissues were sectioned and processed and immunohistochemistry was conducted by Ventana BenchMark GX (Roche, Basel, Switzerland) using anti-ASS1 antibody (1:1000 dilution), anti-DEPTOR antibody (1:250 dilution), and anti-Phospho-S6 Ribosomal Protein (Ser240/244) (1:2000 dilution). The expression of each protein was assessed using a visual grading system on the basis of the intensity of staining signals observed using a light microscope. High intensity (score 3), intermediate intensity (score 2), and low intensity (score 1) were defined as strong, medium, and weak staining, respectively (Fig. 5(a)). H-scores were assigned using the following formula: [1× (% cells of score 1) +2× (% cells of score 2) + 3× (% cells of score 3)]. H-scores of the tumor invasive front and the tumor center were obtained by averaging the H-scores of four random fields of each lesion at 200x magnification and then normalized to the whole H-score of the same specimen. The tumor invasive front was defined as a tumor lesion within 600 μm from the tumor border.

### Immunocytochemistry

Cells were rinsed with ice-cold PBS, fixed with 4% formaldehyde in PBS for 10 min, and permeabilized with 0.05% Triton X-100 in PBS for 1 min. Immunocytochemistry was conducted with the EnVision + kit (Dako, Denmark) according to the manufacturer’s instructions using the anti-ASS1 antibody (1:400 dilution). Cells were counterstained with hematoxylin.

### Immunoblotting

Cells were rinsed three times with ice-cold PBS and lysed in ice-cold lysis buffer (1% NP-40, 10 mM Tris-HCl, 200 mM NaCl, 1 mM EDTA) containing EDTA-free complete protease inhibitor cocktail (Roche) and PhosSTOP (Roche) for 20 min. The soluble fractions from cell lysates were isolated by centrifugation at 13,000 rpm for 10 min in a microcentrifuge. Proteins were analyzed by SDS gel electrophoresis and immunoblotting following standard protocols using the anti-ASS1 antibody (1:200 dilution), anti-p70 S6 Kinase antibody (1:1000 dilution), anti-Phospho-p70 S6 Kinase (Thr389) antibody (1:1000 dilution), anti-DEPTOR/DEPDC6 antibody (1:1000 dilution), anti-Akt antibody (1:1000 dilution), anti-Phospho-Akt (Ser473) antibody (1:2000 dilution), anti-H3K27me3 antibody (1:1000 dilution), anti-Histone H3 antibody (1:1000 dilution), and anti-β-Actin antibody (HRP-conjugated) (1:1000 dilution). Quantification of band intensities was performed by densitometry of membrane images using the Image J software.

### Microarray

Total RNAs were extracted from HEC1B harboring the empty vector (EV) and ASS1-KO HEC1B cells using the miRNeasy kit (Qiagen, Hilden, Germany) according to the manufacturer’s instructions. The quality of the RNA samples was assessed with the Agilent 2100 Bioanalyzer (Agilent Technologies Inc., Palo Alto, CA). Total RNA (200 ng) was reverse-transcribed into double-stranded cDNA using AffinityScript multiple temperature reverse transcriptase (Agilent Technologies Inc.). The resulting complementary RNA (cRNA) was labeled with cyanine-3 (Cy-3) and −5 (Cy-5)-labeled cytosine triphosphate for both cells (Perkin-Elmer, Wellesley, MA) using a Low Input Quick-Amp Labeling Kit (Agilent Technologies Inc.). Two dye-swapped experiments were performed by hybridizing two cRNAs labeled with either Cy-3 or Cy-5 onto a Whole Human Genome Oligo Microarray ver. 2 (G4845A; Agilent Technologies Inc.). The Subio Platform and Subio Basic Plug-in (v1.12; Subio Inc., Aichi, Japan) were then used to calculate the fold change between samples with a two-tailed Student’s t-test using p < 0.05. Microarray data have been deposited in NCBI-GEO under accession numbers GSE86963.

### Real-time PCR

Total RNA was isolated from cells using QIAshredder and RNeasy Mini Kit (Qiagen) and reverse transcription was performed using SuperScript III First-Strand Synthesis System (Thermo Fisher Scientific) according to the manufacturer’s instructions. mRNA transcript levels were measured using the StepOnePlus Real-Time PCR system (Applied Biosystems, Foster City, CA). Data are expressed as the ratio between the expression of DEPTOR and the housekeeping gene ACTB. Human DEPTOR Taqman probe and primers (Applied Biosystems, Hs00961900_m1) and human ACTB Taqman probe and primers (Applied Biosystems, Hs01060665_g1) were used.

### Generation of ASS1-KO cell lines

The *ASS1* gene in HEC1B and AN3CA was disrupted using the CRISPR/Cas9 system^35^. The guide sequences targeting exon 3 and exon 5 of the human *ASS1* gene were designed as described below and cloned into pX330-U6-Chimeric_BB-CBh-hSpCas9 (Addgene, Cambridge, MA). To generate ASS1-KO clones, HEC1B and AN3CA was co-transfected with pX330-U6-Chimeric_BB-CBh-hSpCas9 constructs containing the targeting sequences and the linear puromycin marker (Clontech, Palo Alto, CA) at a weight ratio of 20:1 using Lipofectamine 2000 (Thermo Fisher Scientific) according to the manufacturer’s instructions. One day after transfection, cells were trypsinized and diluted. Then, 1 day after the dilution passage, medium was aspirated and replaced with fresh medium containing 0.25 μg/ml puromycin. When colonies formed, each colony was picked up and expanded, then single-cell clones were established. To confirm that a single-cell clone was an ASS1-knockout clone, the genomic region flanking the targeting sequence was amplified by PCR and subjected to DNA sequencing. The CRISPR targeting sequences used in this study were as follows:

Human *ASS1*:

exon 3: 5′-CACCGACACCTCGTGCATCCTCGTG-3′

exon 5 #1: 5′-CACCGTATGAGGACCGCTACCTCCT-3′

exon 5 #2: 5′-CACCGCTGCATCGCCCGCAAACAAG-3′

The following primers were used to screen for the disrupted alleles:

Human *ASS1*:

exon 3:

(Forward) 5′-TTTGGCGCGCCTGTCTCAGGGTCACTCAGGT-3′

(Reverse) 5′-TTTGCGGCCGCTGGGAAGACTGTGTGCCTTC-3′

exon 5:

(Forward) 5′-TTTGGCGCGCCCCCCTGTCCTTGCCTACTTC-3′

(Reverse) 5′-TTTGCGGCCGCCTCAGAATGGGCGTTCAGGT-3′

Similarly, we transfected the pX330-U6-Chimeric_BBCBh-hSpCas9 empty vector into HEC-1B and AN3CA cells with a linear puromycin marker and constructed a stable cell line, which was used as a control (EV). HEC1B and AN3CA parent cells (PC) were also used as a control.

### Cell proliferation assay

Cells were counted with a Muse Count & Viability Assay Kit and Muse Cell Analyzer (Merck Millipore) according to the manufacturer’s instructions. At the indicated time points, cells were trypsinized and resuspended in medium. A total of 10 μl of cell suspension was added to 190 μl of Muse Count & Viability Reagent and, after 5 min, cells were counted using the Muse Cell Analyzer. Cell proliferation was also assayed by the Premix WST-1 Cell Proliferation Assay System (Takara, Shiga, Japan) according to the manufacturer’s instructions. The absorbance (450 nm and 690 nm) was measured using a SH-9000 Lab microplate reader (Hitachi, Tokyo, Japan).

### Cell migration assay

Cell migration was examined using a scratch assay. Cells were cultured in a 6-cm dish or 6-well plate until they reached 100% confluence to form a monolayer and then a straight line was scraped with a p200 pipette tip. Multiple fields of view per dish or well were imaged at the time of scraping and 24 hours after scraping using a BZ-8000 microscope (KEYENCE, Osaka, Japan). The distances between cells were measured using Image J software. In arginine or lysine depletion experiments, cells were cultured in arginine- or lysine-depleted DMEM with 10% dialyzed FBS for 96 hours. After scraping, the medium was replaced by DMEM containing 400 μM arginine or 800 μM lysine with 10% dialyzed FBS. Multiple fields of view per dish or well were imaged at the time of scraping and 24 hours after scraping using a BZ-8000 microscope (Fig. 2(a) and see Supplementary Fig. S6(a)). When Torin1 was used, cells were incubated with 100 nM Torin1 after scraping. In arginine depletion experiments after transfection of a DEPTOR expressing vector, an ASS1 expressing vector, or an empty control vector, the medium was replaced by arginine-depleted DMEM with 10% dialyzed FBS 12 hours after transfection and cells were cultured for 96 hours in arginine-depleted condition. After scraping, the medium was replaced by DMEM containing 400 μM arginine with 10% dialyzed FBS. Multiple fields of view per dish or well were imaged at the time of scraping and 24 hours after scraping using a BZ-8000 microscope.

### Cell invasion assay

Cell invasion was examined using Corning BioCoat Matrigel Invasion Chambers with 8.0 μm PET Membrane (Corning, New York City, NY). Cells (1 × 10^5^) in serum-free DMEM were seeded in the upper chamber and 10% FBS-DMEM was added in the lower chamber. In arginine depletion experiments, after 96 hours of arginine starvation, cells (1 × 10^5^) in serum-free and arginine-depleted DMEM were seeded in the upper chamber, and 10% dialyzed FBS-DMEM with or without arginine (final concentration 400 μM) was added in the lower chamber (Fig. 2(a)). After 24 hours of incubation, cells on the top surface of the insert were removed by wiping with a cotton swab. The cells that had invaded the bottom side of the membrane were fixed and stained using Diff-Quik stain (Sysmex, Hyogo, Japan). The numbers of invaded cells were obtained by averaging four random fields at 200x magnification per membrane and the average of three membranes is presented.

### Bisulfite sequencing

Genomic DNA from EV and ASS1-KO HEC1B cells was bisulfite-converted with the EpiTect Fast DNA Bisulfite Kit (Qiagen) according to the manufacturer’s instructions. After bisulfite conversion, DNA was amplified with Takara EpiTaq HS (Takara). The following primers were used to amplify DNA:

(Forward) 5′-TTTGGCGCGCCTTGTTTATTTATAGGGATTTTTTTT-3′

(Reverse) 5′-TTTGCGGCCGCCACTACCAACACTACCAATACTACC-3′

PCR products were purified using the Gel Extraction Kit (Qiagen). Purified PCR products were cloned into the pEGFP3 vector. Fifteen clones were sequenced from each sample.

Chromatin immunoprecipitation (ChIP) assay

ChIP assays in EV and ASS1-KO cells were performed using the SimpleChIP Enzymatic Chromatin IP Kit (Cell Signaling Technology) and antibodies to H3K4me3 (Cell Signaling Technology, #9751), H3K9me2 (Abcam, ab1220), and H3K27me3 (Abcam, ab6002). Immunoprecipitations using IgG (Cell Signaling Technology, #2729) were performed as negative controls. Purified DNA was analyzed by quantitative real-time PCR using the StepOnePlus Real-Time PCR system (Applied Biosystems) and SYBR Premix Ex Taq II (Takara). The following primers were used in PCR amplifications:

350 bp downstream relative to the *DEPTOR* transcriptional start site:

(Forward) 5′-TAAGGCAAGAGACACAGCGG-3′

(Reverse) 5′-GCACCGAGGACCATCCAC-3′

700 bp downstream relative to the *DEPTOR* transcriptional start site:

(Forward) 5′-ATCCGCGAAGAGAGCAAGAG-3′

(Reverse) 5′-GCTCAGGGGAGTGAAGGAAG-3′

Mbp upstream relative to the *DEPTOR* transcriptional start site:

(Forward) 5′-GCTGTTTGGCTGGCTTGAAA-3′

(Reverse) 5′- ATCTGCTTGGCATCCTTGGT-3′

### Statistical analysis

The database was created using Microsoft Excel and analyzed using JMP Pro 12 (SAS Institute, Cary, NC). The normality of data was evaluated by Shapiro-Wilk test at the significant level of 0.05. When the normality is confirmed in the groups, the homogeneity of variance was tested by F-test at the significant level of 0.05. When two groups were normally distributed with or without equal variance, Student’s t-test or Welch’s t-test was applied, respectively. When at least one of the two groups was not normally distributed, Mann-Whitney U test was applied. In densitometry analysis, one sample t-test or Student’s t-test was applied after confirming the normality and equal variance by Shapiro-Wilk test and F-test. Assessment of differences between H-scores among endometrioid carcinoma specimens was performed using Welch’s t-test. For all boxplots, boxes depict the middle 50% of the records and the line indicates the median. Whiskers show the highest and lowest values. Statistical significance was set as P < 0.05, two-tailed.

## Additional Information

**How to cite this article**: Ohshima, K. *et al*. Argininosuccinate Synthase 1-Deficiency Enhances the Cell Sensitivity to Arginine through Decreased DEPTOR Expression in Endometrial Cancer. *Sci. Rep.* **7**, 45504; doi: 10.1038/srep45504 (2017).

**Publisher's note:** Springer Nature remains neutral with regard to jurisdictional claims in published maps and institutional affiliations.

## Supplementary Material

Supplementary Figures and Table
