# Supplementary material for: Argininosuccinate Synthase 1-Deficiency Enhances the Cell Sensitivity to Arginine through Decreased DEPTOR Expression in Endometrial Cancer
Source: Sci Rep. 2017 Mar 30;7:45504. doi: 10.1038/srep45504 (PMC5371991; doi:10.1038/srep45504)
Supplement: Supplementary Figures and Table [file srep45504-s1.pdf]

Title

Argininosuccinate Synthase 1-Deficiency Enhances the Cell Sensitivity to Arginine through Decreased DEPTOR Expression in Endometrial Cancer

Authors

Kenji Ohshima[1], Satoshi Nojima[1], Shinichiro Tahara[1], Masako Kurashige[1], Yumiko Hori[1], Kohei Hagiwara[2], Daisuke Okuzaki[3], Shinya Oki[4], Naoki Wada[1], Jun-ichiro Ikeda[1], Yoshikatsu Kanai[2], and Eiichi Morii[1*]

[1] Department of Pathology, Osaka University Graduate School of Medicine, Osaka, Japan.

[2] Department of Bio-system Pharmacology, Osaka University Graduate School of Medicine, Osaka, Japan.

[3] Department of Molecular Genetics, Research Institute for Microbial Diseases, Osaka University, Osaka, Japan.

[4] Department of Developmental Biology, Graduate School of Medical Sciences, Kyushu University, Fukuoka, Japan.

*Correspondence to E. Morii

morii@molpath.med.osaka-u.ac.jp

Supplementary Figure S1

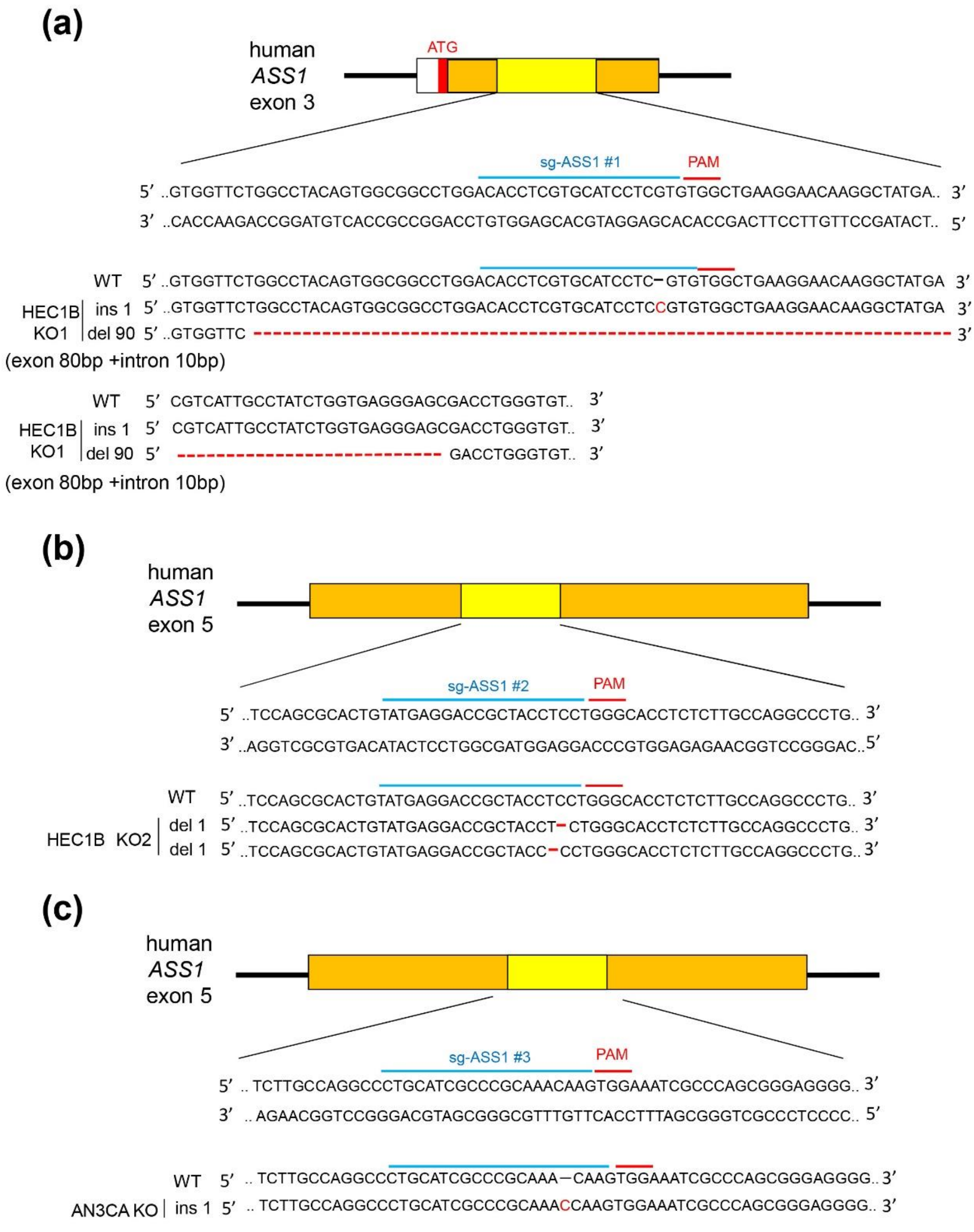

Supplementary Figure S1. Generation of CRISPR/CAS9-mediated ASS1 KO HEC1B and AN3CA cells

(a and b) A schematic of the single-guide (sg)RNA-targeting sites in the human *ASS1* gene. Two targeting sequences were used in this study: ASS1 (a) #1, (b) #2 and (c) #3. The (a) third and (b),(c) fifth exons of the human *ASS1* gene are shown as a box. White, 5′ UTR; Orange, coding sequence. Targeting sites and protospacer adjacent motifs (PAMs) are indicated as blue and red bars, respectively. Sequence alignments of the WT *ASS1* gene and the disrupted alleles from ASS1-KO HEC1B clone (a) HEC1B KO1, (b) HEC1B KO2 and (c) AN3CA KO are shown. Inserted nucleotides are highlighted in red. Deleted regions are indicated with red dashes. Del X, deletion of X base pair (bp); ins X, insertion of X bp. One allele of (a) KO1 had a deletion of an 80-bp exon and 10-bp intron.

Supplementary Figure S2

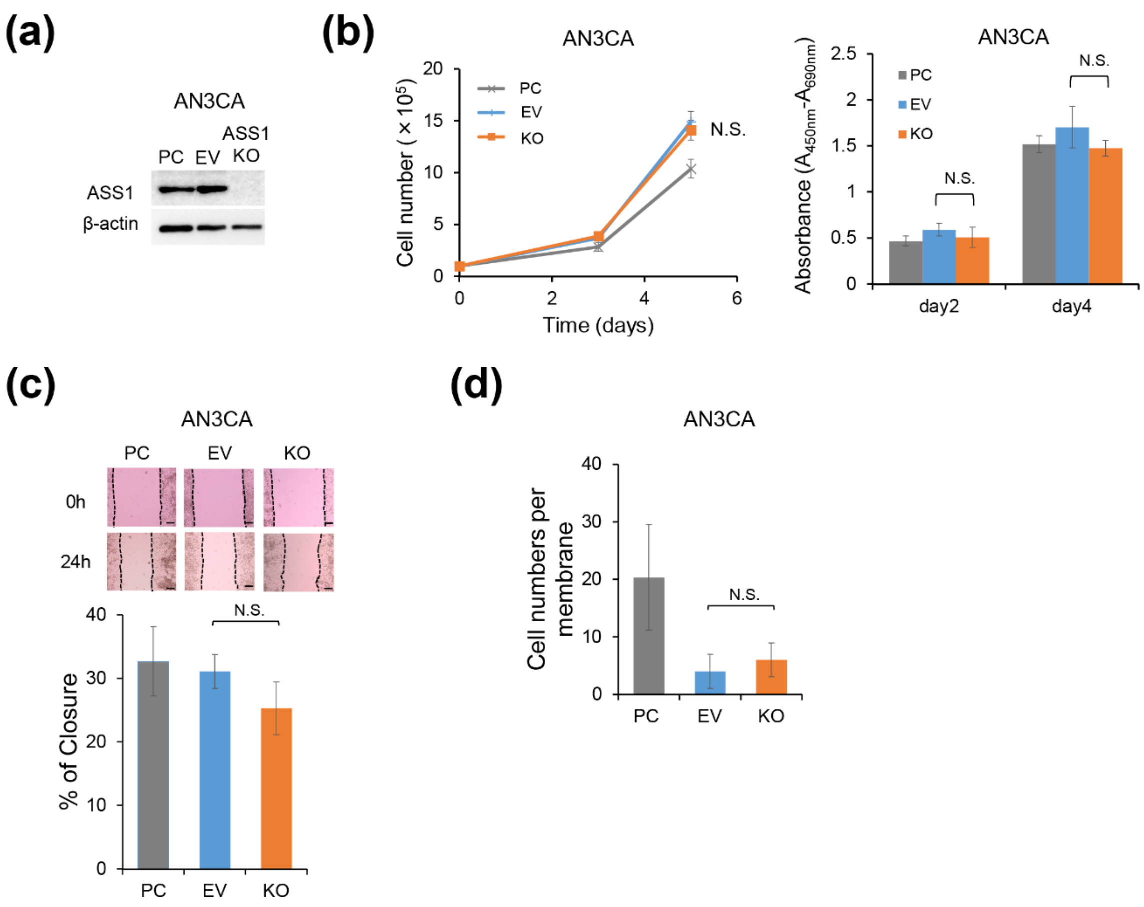

Supplementary Figure S2. ASS1 deficiency does not affect proliferation, motility, and invasion of AN3CA cells when cultured in DMEM containing arginine.

(a) Immunoblotting of ASS1 in PC, EV, and ASS1-KO AN3CA cells generated using the CRISPR/Cas9 system. (b)-(d) When cells were cultured in DMEM prepared for standard culture conditions (complete DMEM), there was no significant difference in (b) cell proliferation (n=3) assessed by cell counting (left panel) and WST-1 assay (right panel). A total of $1{\times}10^5$ cells were seeded into a 6-well plate for the cell counting assay and $5{\times}10^3$ cells were seeded into a 96-well plate for the WST-1 assay. Data are representative of three

independent experiments. (c) Cell motility (n=3) assessed using a scratch assay, and (d) invasion ability (n=4) assessed using a Transwell Matrigel invasion assay. Data are representative of three independent experiments. Data are shown as mean ± SD. Scale bars, 400 μm in (c), 200 μm in (d). N.S., not significant, Student's t-test.

Supplementary Figure S3

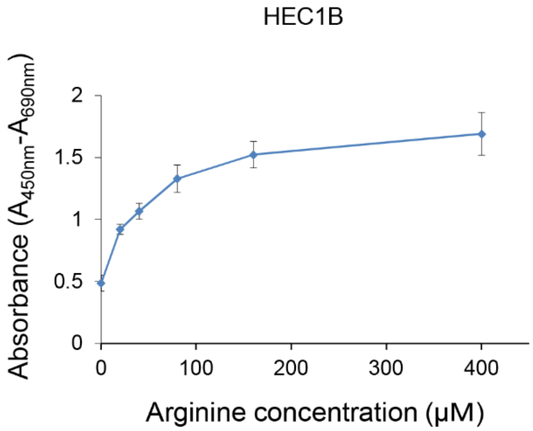

Supplementary Figure S3. Proliferation of HEC1B cells declined in proportion to extracellular arginine concentration

HEC1B cells (1.5×10^3 cells) were resuspended in 100 μl of DMEM and seeded into a 96-well plate. Twelve hours after seeding, the medium was replaced with DMEM with 10% dialyzed FBS containing the indicated concentration of arginine. Four days after medium replacement, WST-1 assay was conducted (n=3). Data are representative of three independent experiments. Data are shown as mean ± SD.

Supplementary Figure S4

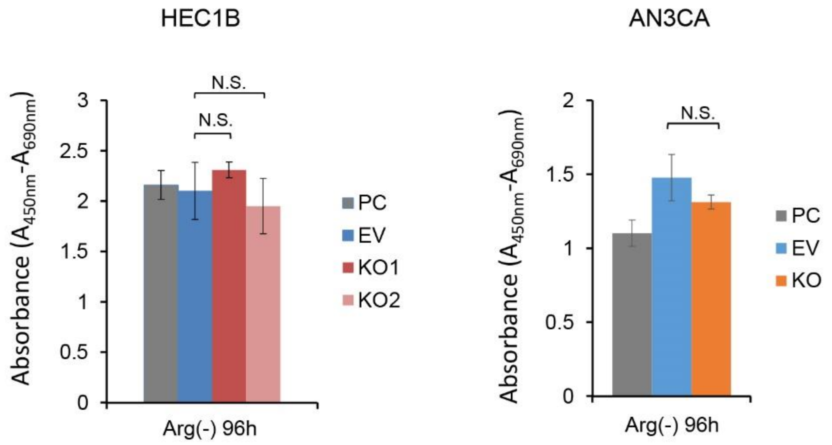

Supplementary Figure S4. There is no significant difference in proliferation between PC, EV, and ASS1-KO cells when cultured in arginine-depleted DMEM

PC, EV, and ASS1-KO HEC1B cells (1×10^4 cells) (left panel) and PC, EV, and ASS1-KO AN3CA cells (1×10^4 cells) (right panel) were resuspended in 100 µl of DMEM and seeded into a 96-well plate. Twelve hours after seeding, medium was replaced with arginine-depleted DMEM with 10% dialyzed FBS. Four days after medium replacement, a WST-1 assay was conducted (n=3). Data are representative of three independent experiments. Data are shown as mean ± SD. N.S., not significant, Student's t-test.

.

Supplementary Figure S5

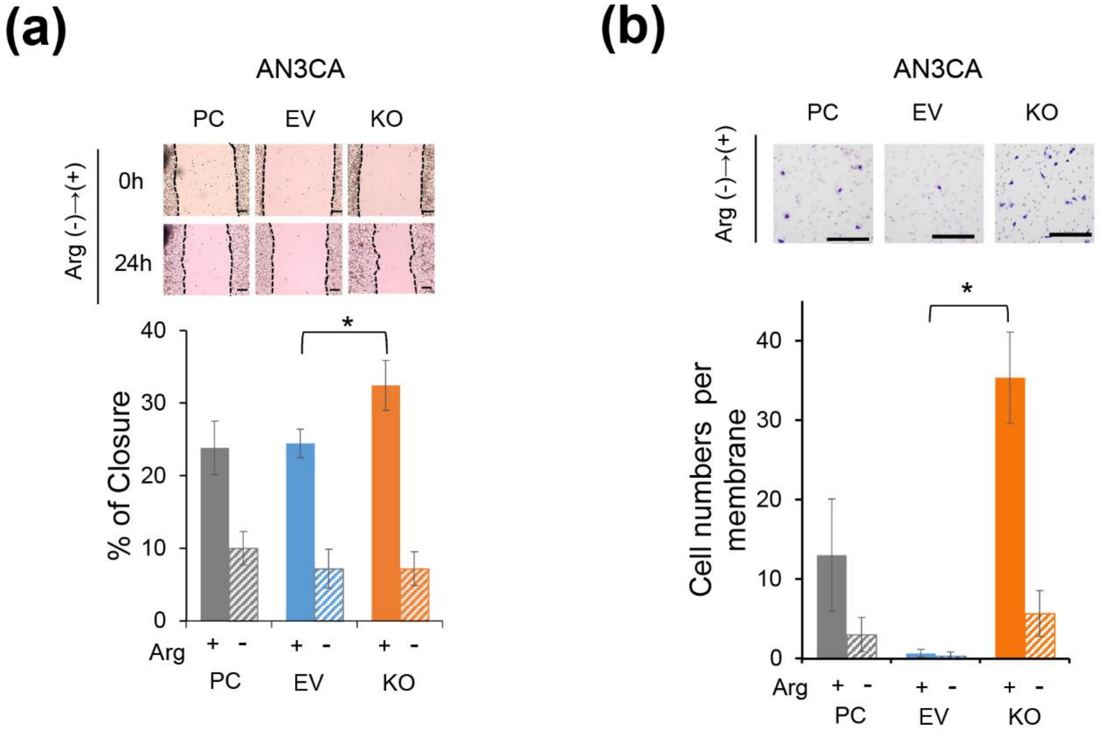

Supplementary Figure S5. ASS1 deficiency enhances the cell motility and invasion capability of AN3CA cells in response to re-supplementation with arginine following arginine starvation.

(a) Cell motility of PC, EV, or ASS1-KO AN3CA cells was assessed by scratch assay (n=3). Data are representative of three independent experiments. Data are shown as the mean ± SD. Scale bars, 400 μm * $p < 0.05$, Student's t-test. (b) Invasion capability of PC, EV, or ASS1-KO AN3CA cells was assessed by Transwell Matrigel invasion assay (n=4). Because AN3CA cells showed much lower invasion capability than HEC1B cells, we counted total cell numbers in the whole membranes. Data are representative of four independent

experiments. Data are shown as the mean ± SD. Scale bars, 200 μm * $p < 0.05$, Mann-Whitney U test.

Supplementary Figure S6

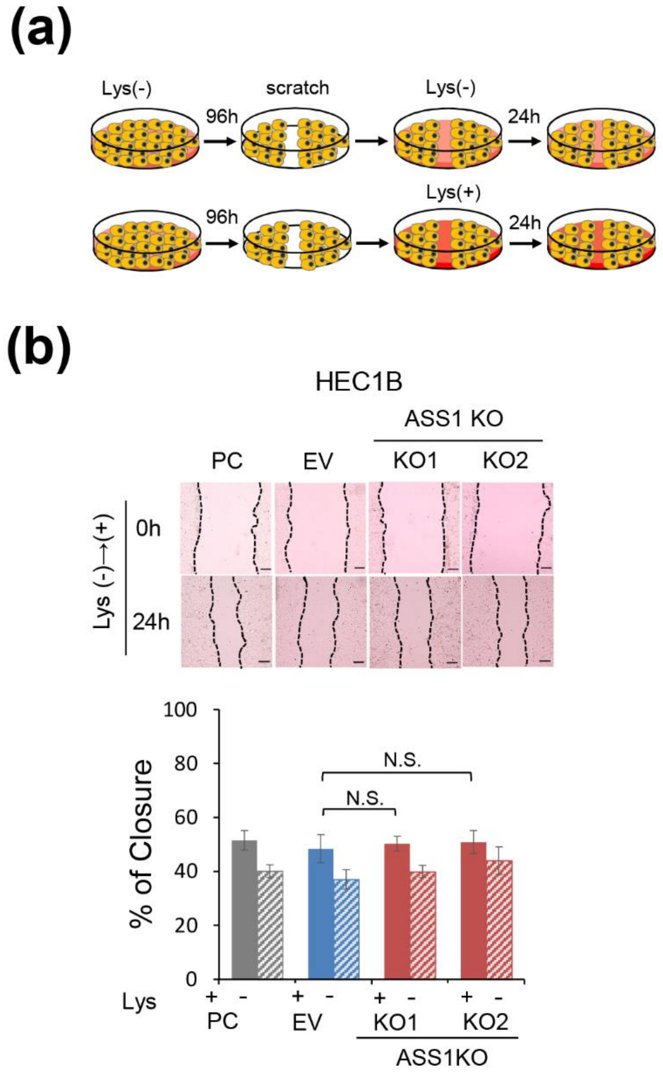

Supplementary Figure S6. Lysine depletion does not enhance motility of ASS1-KO HEC1B cells

(a) Schematic experimental procedure of the scratch assay following lysine depletion. (b) There was no significant difference in cell motility (n=4) between PC, EV, and ASS1-KO

HEC1B cells when re-supplemented with lysine (800 μM) following 96-hour lysine starvation. Scale bars, 400 μm. Data are shown as mean ± SD. N.S., not significant, Student's t-test.

Supplementary Figure S7

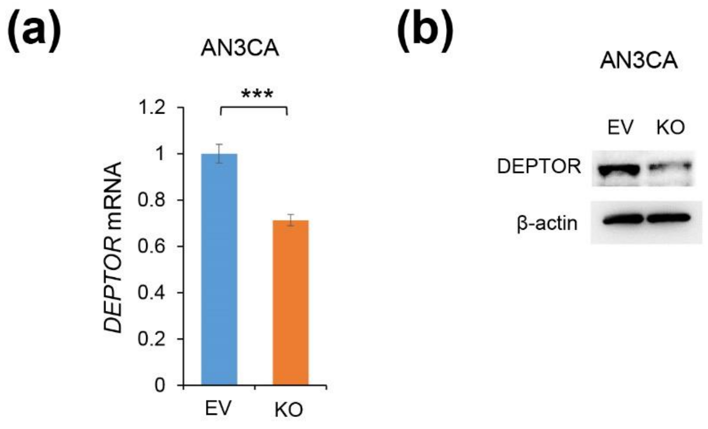

Supplementary Figure S7. DEPTOR mRNA and protein expression levels were decreased in ASS1-KO AN3CA cells compared with those in EV AN3CA cells

(a) *DEPTOR* mRNA was evaluated by real-time PCR. Data are representative of two independent experiments. Data are shown as mean ± SD. *** $p < 0.001$, Student's t-test. (b) DEPTOR protein expression were evaluated by immunoblotting. Data are representative of three independent experiments.

Supplementary Figure S8

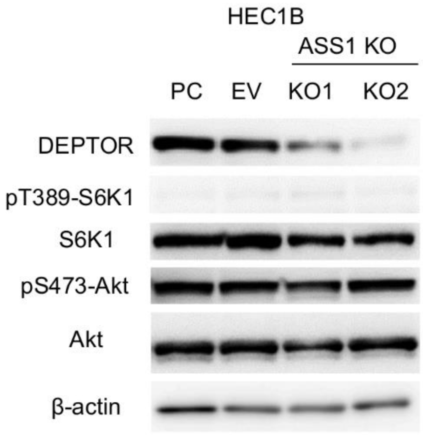

Supplementary Figure S8. DEPTOR protein expression decreases in ASS1-KO HEC1B cells even under arginine-depleted conditions

After culture in arginine-depleted condition for 96 hours, PC, EV, and ASS1-KO HEC1B cells were lysed for immunoblotting. In the same way as for the arginine-replete conditions, ASS1-KO HEC1B showed lower DEPTOR protein expression than PC and EV under arginine-depleted conditions, while mTORC1 and mTORC2 activities did not differ among them.

Supplementary Figure S9

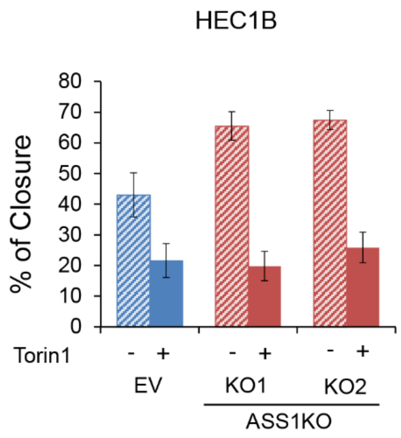

Supplementary Figure S9. mTOR inhibitor Torin1 eliminates higher cell motility of ASS1-KO HEC1B cells

Treatment with Torin1 (100 nM) significantly suppressed the motility of ASS1-KO HEC1B cells to the same level as in EV cells, when cells were re-supplemented with arginine (400 µM) following 96-hour arginine starvation. Data are representative of three independent experiments. Data are shown as the mean ± SD.

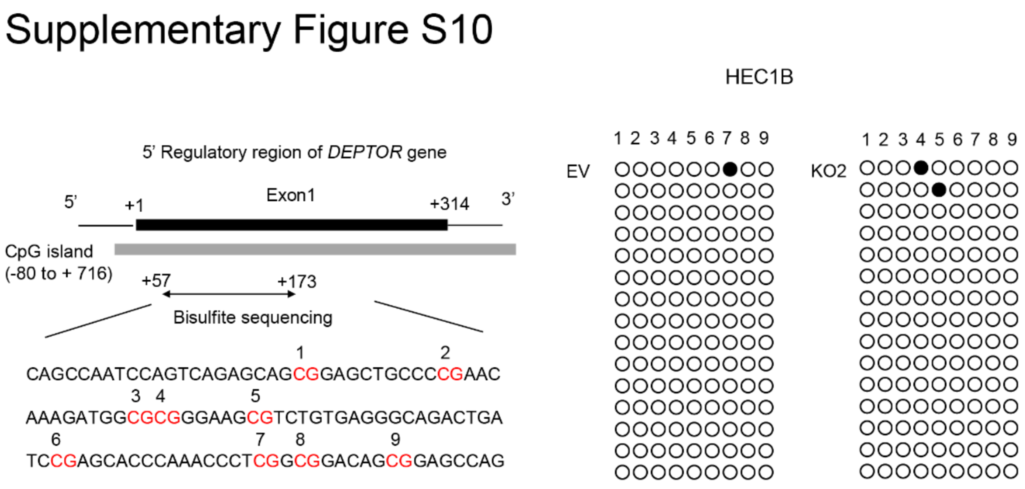

Supplementary Figure S10. DNA methylation at the *DEPTOR* promoter region of EV and ASS1-KO HEC1B cells

Bisulfite sequencing of the *DEPTOR* CpG islands. Left, Schematic depiction of the DEPTOR CpG islands, spanning the region from −80 to +716 (with the transcription start site at +1). Regions analyzed by bisulfite sequencing are shown. Right, Bisulfite sequencing results of EV and ASS1-KO HEC1B. Each clone is represented by a row, and the CG dinucleotides being investigated are arranged in columns. White and black circles represent unmethylated and methylated cytosines, respectively.

Supplementary Figure S11

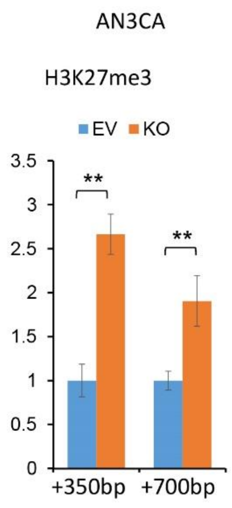

Supplementary Figure S11. H3K27me3 in the *DEPTOR* promoter region of ASS1-KO AN3CA cells

ChIP-qPCR analysis of the 5′ regulatory region of DEPTOR in EV and ASS1-KO AN3CA cells using antibodies to H3K27me3 (n=3). The locations of the qPCR primers are relative to the transcriptional start site of DEPTOR. Data are expressed as a ratio relative to the percent of input of EV. Data are representative of two independent experiments. Data are shown as mean ± SD. ** $p < 0.01$, Welch's's t-test.

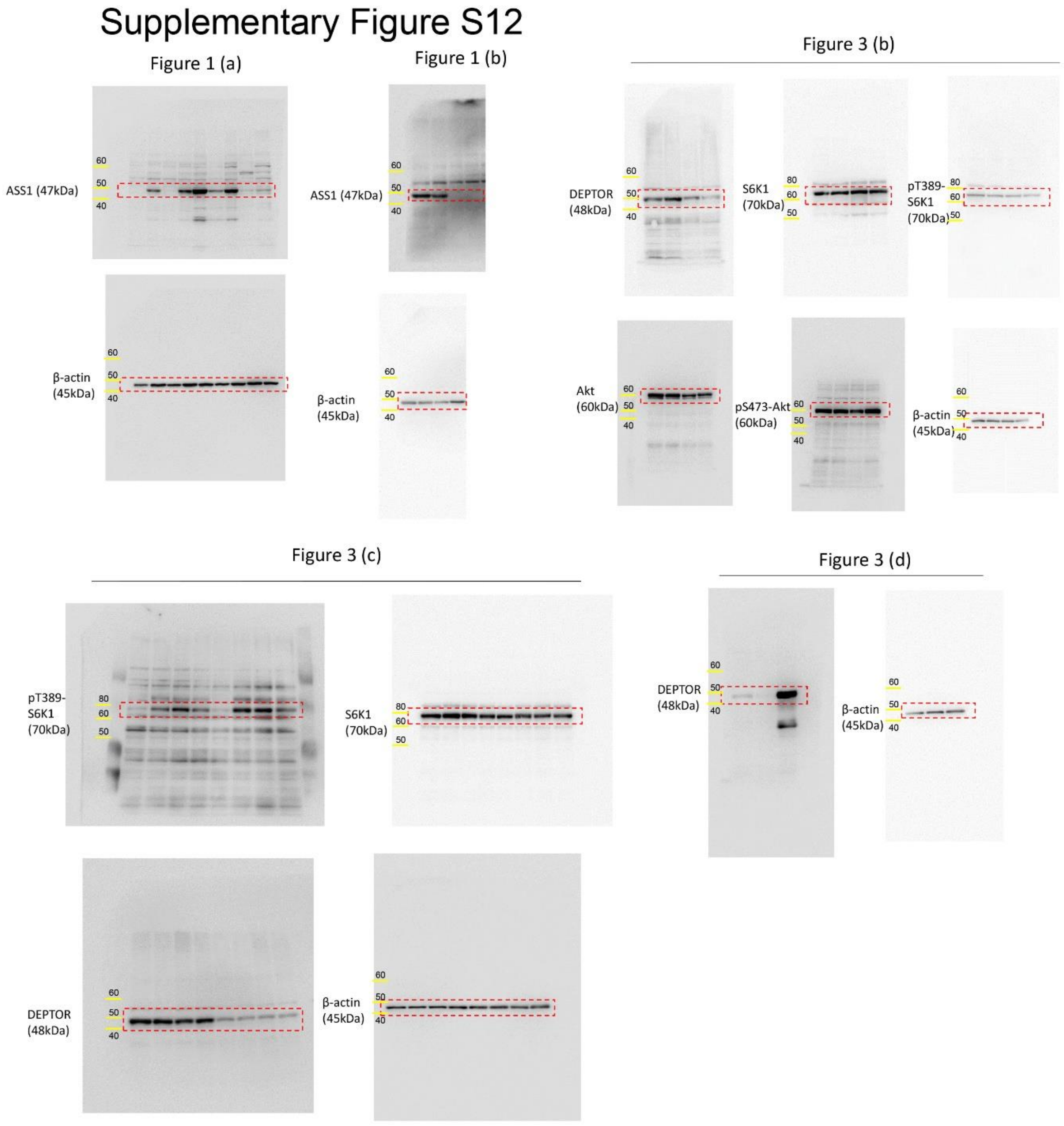

Supplementary Figure S12. Full-length blots in the main paper are presented.

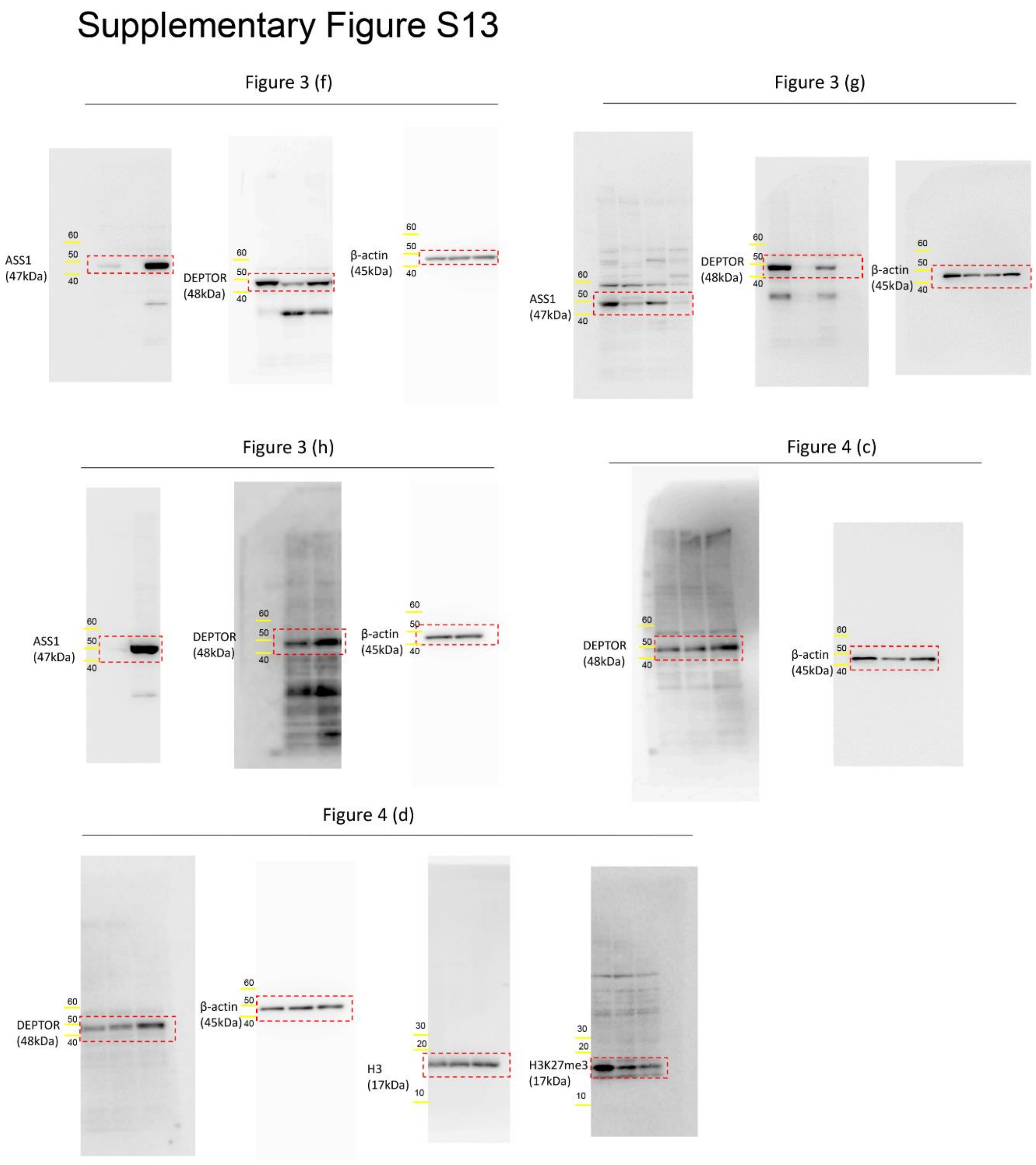

Supplementary Figure S13. Full-length blots in the main paper are presented.

Supplementary Table S1

	Number of cases
age (years)	
≧60	28
< 60	46
Histological grade	
G1	22
G2	26
G3	26
T stage	
T1	53
T2	6
T3	15
N stage	
N0	63
N1	11
M stage	
M0	71
M1	3

Supplementary Table S1. The clinicopathological characteristics of the patients

T, N, and M stage are according to *UICC TNM Classification of Malignant Tumours, 7th edition*.
